# Greater tau load and reduced cortical thickness in *APOE* ε4-negative Alzheimer’s disease: a cohort study

**DOI:** 10.1186/s13195-018-0403-x

**Published:** 2018-08-07

**Authors:** Niklas Mattsson, Rik Ossenkoppele, Ruben Smith, Olof Strandberg, Tomas Ohlsson, Jonas Jögi, Sebastian Palmqvist, Erik Stomrud, Oskar Hansson

**Affiliations:** 1 Clinical Memory Research Unit, Department of Clinical Sciences, Faculty of Medicine, Lund University, Lund, Sweden; 2 Department of Neurology, Skåne University Hospital, Lund University, Lund, Sweden; 3 VU University Medical Center, Neuroscience Campus Amsterdam, Amsterdam, The Netherlands; 4 Department of Radiation Physics, Skåne University Hospital, Lund, Sweden; 5 Department of Clinical Physiology and Nuclear Medicine, Skåne University Hospital, Lund, Sweden; 6 Memory Clinic, Skåne University Hospital, Malmö, Sweden

**Keywords:** APOE, Tau, Atrophy, Cognition, Alzheimer’s disease

## Abstract

**Background:**

Alzheimer’s disease is characterized by aggregated β-amyloid and tau proteins, but the clinical presentations and patterns of brain atrophy vary substantially. A part of this heterogeneity may be linked to the risk allele *APOE* ε4. The spread of tau pathology is related to atrophy and cognitive decline, but little data exist on the effects of *APOE* ε4 on tau. The objective of this preliminary study was therefore to test if tau load and brain structure differ by *APOE* ε4 in Alzheimer’s disease.

**Methods:**

Sixty-five β-amyloid-positive patients at the prodromal and dementia stages of Alzheimer’s disease were enrolled, including *APOE* ε4-positive (*n* = 46) and *APOE* ε4-negative (*n* = 19) patients. ^18^F-AV-1451 positron emission tomography was used to measure tau and brain magnetic resonance imaging (MRI) was used to measure cortical thickness.

**Results:**

Compared with their *APOE* ε4-positive counterparts, *APOE* ε4-negative patients had greater tau load and reduced cortical thickness, with the most pronounced effects for both in the parietal cortex. Relative to the overall cortical tau load, *APOE ε4*-positive patients had greater tau load in the entorhinal cortex. *APOE* ε4-positive patients also had slightly greater cortical β-amyloid load. There was an interaction between *APOE* ε4 and ^18^F-AV-1451 on cortical thickness, with greater effects of ^18^F-AV-1451 on cortical thickness in *APOE* ε4-negative patients. *APOE* ε4 and ^18^F-AV-1451 were independent predictors of cognition, but showed distinct associations with different cognitive tests.

**Conclusions:**

*APOE* genotype may be associated with differences in pathways in Alzheimer’s disease, potentially through differential development and spread of tau, as well as through effects on cognitive outcomes involving non-tau-related mechanisms.

**Electronic supplementary material:**

The online version of this article (10.1186/s13195-018-0403-x) contains supplementary material, which is available to authorized users.

## Background

Most Alzheimer’s disease (AD) patients have an amnestic-predominant cognitive impairment profile, while others have more prominent executive, language, or visuospatial deficits [1, 2]. The variations in presentations may be associated with specific patterns of neurodegeneration or tau pathology [3–6], but the reason why the regional involvement of neurodegeneration and tau varies is largely unclear [7]. One possibility is that the apolipoprotein (*APOE*) ε4 allele contributes to the variability. *APOE* ε4 is a major genetic risk factor for sporadic AD, and is present in 50–70% of patients [8–10]. However, roughly a third of all AD patients develop the disease without carrying an *APOE* ε4 allele. *APOE* ε4-negative patients are characterized by relatively more nonamnestic deficits and anatomically by greater frontoparietal atrophy, while *APOE* ε4-positive patients predominantly demonstrate memory impairment and temporal lobe atrophy [11–15]. Given the intimate link between tau pathology and neurodegeneration in neuroimaging [3, 16–19], neuropathology [20], and cell and animal studies [21], it is possible that *APOE* ε4 status affects the spread of tau in AD and subsequent brain atrophy, but only few studies have explored this [22–24]. We tested if *APOE* ε4 was associated with regional and global differences in ^18^F-AV-1451 in prodromal AD and AD dementia patients, and if *APOE* ε4 status and ^18^F-AV-1451 interacted to predict atrophy and cognition. We hypothesized that *APOE* ε4-positive patients would have more tau pathology and atrophy in the temporal lobe, while *APOE* ε4-negative patients would be more affected by tau and atrophy in other brain regions, in particular the frontal and parietal areas.

## Methods

### Participants

All participants were recruited from the Swedish BioFINDER study. Inclusion and exclusion criteria have been described previously [25]. We included prodromal AD (amyloid-β (Aβ)-positive mild cognitive impairment [MCI]) [26] and mild-to-moderate (Aβ-positive) AD dementia patients who were all assessed by physicians with expertise in dementia. Aβ-positivity was defined by cerebrospinal fluid (CSF) Aβ42 in all cases except one for whom ^18^F-flutemetamol positron emission tomography (PET) was used. The inclusion criteria for MCI were: referred to a memory clinic due to possible cognitive impairment; objective impairment in one or more cognitive domains; preservation of independence in functional abilities; and not fulfilling criteria for any dementia disorder. All dementia patients met the NIA-AA criteria for dementia due to AD [27]. The exclusion criteria were: cognitive impairment that without doubt could be explained by another condition other than prodromal AD or AD dementia; significant systemic illness making it difficult to participate; and significant alcohol or drug abuse.

### Cognitive measures

Cognitive measures included Mini-Mental State Examination (MMSE) [28] for global cognition, the immediate and delayed conditions of the 10-word list recall tests from the Alzheimer’s Disease Assessment Scale (ADAS)-cognitive subscale [29] for memory, Trail Making Test-A (TMT-A) [30], and A Quick Test of cognitive speed—Color & Form subtest (AQT-CF) [31] for attention and processing speed, and category fluency [30] for language and semantic memory (animal fluency) and executive function (letter S fluency). Data were missing for MMSE in one subject, for ADAS immediate recall in six subjects, for ADAS delayed recall in seven subjects, for TMT-A in nine subjects, for letter S fluency and animal fluency in 12 subjects, and for AQT-CF in 14 subjects. The reason for missing cognitive data was either refusal or cognitive inability to perform the task. We kept all eligible subjects for this analysis, even if they lacked some cognitive data, since exclusion based on missing data would have increased the risk of a selection bias.

### CSF biomarkers

Lumbar CSF sampling was performed following the Alzheimer’s Association Flow Chart [32]. Samples were stored in 1-ml polypropylene tubes at −80 °C until analysis. Enzyme-linked immunosorbent assays (ELISAs) were used for CSF Aβ42, total (T)-tau, and phosphorylated (P)-tau (ADx/Euroimmun AG, Lübeck, Germany, and Innotest, Fujirebio, Ghent, Belgium). All analyses were performed by board-certified laboratory technicians who were blinded for clinical data and diagnoses. Aβ-positivity was defined as CSF Aβ42 < 647 ng/L [33]. For one subject who refused to undergo lumbar puncture we determined Aβ-positivity by ^18^F-flutemetamol PET (see below for details).

### Magnetic resonance imaging (MRI)

T1-weighted MRI was performed on a 3-T MR scanner in all subjects (Siemens Tim Trio 3 T, Siemens Medical Solutions, Erlangen, Germany), producing a high resolution anatomical MP-RAGE image (TR = 1950 ms, TE = 3.4 ms, 1 mm isotropic voxels, and 178 slices). Cortical reconstruction and volumetric segmentation were performed with FreeSurfer (v5.3) (http://surfer.nmr.mgh.harvard.edu/) using an in-house developed image analysis pipeline. The MP-RAGE images underwent correction for intensity homogeneity [34], removal of nonbrain tissue, and segmentation into gray matter (GM) and white matter (WM) with intensity gradient and connectivity among voxels [35–38]. Cortical thickness was measured as the distance from the GM/WM boundary to the corresponding pial surface [36]. Reconstructed datasets were visually inspected for accuracy, and segmentation errors were corrected. The surface area-weighted average cortical thickness was calculated for seven FreeSurfer-based metaregions: lateral parietal, medial parietal, lateral temporal, medial temporal, frontal, occipital, and whole brain cortex (Additional file 1: Table S1). We also calculated the ratio between entorhinal cortical thickness and overall cortical thickness in accordance with Whitwell et al. [24] (E/C ratio, the overall cortical region included the whole cortex except for entorhinal and the inferior temporal gyri, since tau accumulation there may be closely linked to entorhinal tau).

Automated segmentation of white matter lesions (WML) was performed using the lesion prediction algorithm as implemented in the LST toolbox (www.statistical-modelling.de/lst.html) for SPM. This algorithm consists of a binary classifier in the form of a logistic regression model; as covariates, a lesion belief map [39] is used as well as a spatial covariate that takes into account voxel-specific changes in lesion probability. Parameters of this model fit are used to segment lesions by providing an estimate for the lesion probability for each voxel.

### ^18^F-AV-1451 tau PET imaging and processing

Tau PET was performed in all subjects as described previously [40]. Briefly, ^18^F-AV-1451 was synthesized at Skåne University Hospital, Lund [41], and PET scans were performed on a GE Discovery 690 PET scanner (General Electric Medical Systems). FreeSurfer parcellation in the MR space of the anatomical scan was applied to processed, coregistered and time-averaged PET images to extract regional uptake values. ^18^F-AV-1451 standardized uptake value ratio (SUVr) images were based on mean uptake over 80–100 min postinjection normalized to uptake in a GM-masked inferior cerebellum reference region. The signal was not corrected for partial volume effects.

The same FreeSurfer metaregions as for MRI, including the E/C ratio, were calculated for ^18^F-AV-1451 (volume weighted; Additional file 1: Table S1). We excluded the hippocampus from the medial temporal metaregion because of its susceptibility to spill-over effects from the anatomically proximate choroid plexus [40].

### ^18^F-flutemetamol PET imaging

Fibrillary brain Aβ was quantified in a subgroup (*n* = 54) using ^18^F-flutemetamol PET. PET/computed tomography (CT) scanning was conducted at two sites using the same type of scanner, a Philips Gemini TF 16. PET sum images from 90 to 110 min postinjection were generated for the average uptake. FreeSurfer parcellation in the MR space of the anatomical scan was applied to the processed images. The SUVr images were normalized to the mean uptake in a composite region consisting of cerebellar white matter, brainstem and cerebral white matter. We used the same FreeSurfer metaregions (volume weighted) as for ^18^F-AV-1451 and additionally assessed the striatum.

^18^F-flutemetamol PET SUVr in a composite neocortical region-of-interest indicated Aβ-positivity in one subject who refused to undergo lumbar puncture [42].

### Statistical analysis

Statistical analyses were performed with R (v. 3.3.2, The R Foundation for Statistical Computing). The relationships between demographics and *APOE* ε4 were evaluated with Fisher’s exact test and Wilcoxon-Mann-Whitney rank sum test.

We used linear regression to test effects of *APOE* ε4 status on ^18^F-AV-1451, ^18^F-flutemetamol, cortical thickness, and interactions of *APOE* ε4 status and ^18^F-AV-1451 to predict cortical thickness and cognition. Covariates included age, sex, education, CSF Aβ42, WML, and CSF P-tau.

In secondary analyses, we compared models for *APOE* ε4 and ^18^F-AV-1451 to predict cognition, with and without adjusting for each other, to test if they provided independent information. We adjusted for brain MRI measures to test if effects of *APOE* ε4 and ^18^F-AV-1451 on cognition were independent of atrophy. Akaike Information Criterion (AIC) was used for model comparisons. Statistical significance was determined at *P* < 0.05.

### Primary research question

Does regional cortical thickness and ^18^F-AV-1451 PET uptake differ by *APOE* ε4 status in AD patients at the prodromal and dementia stage of the disease?

## Results

We included 46 *APOE* ε4-positive and 19 *APOE* ε4-negative AD patients, with no significant group differences regarding age, sex, education, WML, hippocampal volume, global cognition (MMSE), or CSF Aβ42 (Table 1). *APOE* ε4-negative patients had higher CSF T-tau (*P* = 0.035) and P-tau (*P* = 0.012), better memory function (the ADAS delayed recall, *P* = 0.018), and worse executive function (TMT-A, *P* = 0.021). Fifty-four patients (37 *APOE* ε4-positive and 17 *APOE* ε4-negative) had ^18^F-flutemetamol PET data. The *APOE* ε4-positive patients had slightly greater Aβ load (Additional file 1: Figure S1).

**Table 1:** Patient characteristics

	*APOE* ε4-negative (*n* = 19)	*APOE* ε4-positive (*n* = 46)	*P* value
Diagnosis, *n* (MCI due to AD/AD dementia)	4/15	18/28	0.27
Age (years)	70.1 ± 7.8	72.4 ± 6.8	0.23
Sex, *n* (male/female)	9/10	18/28	0.29
Education, years	11.1 ± 2.7	12.5 ± 3.4	0.13
*APOE* genotype	ε3/ε3 (*n* = 19)	ε2/ε4 (*n* = 3) ε3/ε4 (*n* = 27) ε4/ε4 (*n* = 16)	NA
CSF Aβ42, ng/L	405 ± 97	405 ± 104	0.99
**CSF T-tau, ng/L**	**855 ± 366**	**673 ± 283**	**0.035**
**CSF P-tau, ng/L**	**105 ± 4**	**81 ± 31**	**0.012**
WML, mL	15.5 (13.8)	11.9 (14.0)	0.31
Hippocampal volume, mL	2968 (576)	2907 (442)	0.50
MMSE	22.2 ± 5.8	22.6 ± 4.3	0.73
ADAS immediate recall	5.9 ± 1.8	5.9 ± 1.3	0.98
**ADAS delayed recall**	**6.2 ± 3.1**	**8.0 ± 2.01**	**0.018**
**TMT-A**	**123.6 ± 112.0**	**75.0 ± 42.5**	**0.021**
AQT-CF	102.9 ± 27.6	91.2 ± 38.5	0.35
Fluency—animals	9.9 ± 6.8	12.1 ± 5.2	0.21
Fluency—letter (S)	8.0 ± 5.6	11.1 ± 4.9	0.055

Continuous data are shown as mean and standard deviations *P* values are from chi-square tests and linear regression models; significant results (*P* < 0.05) are indicated in bold. Hippocampal volume was averaged between right and left. Tests for WML and hippocampal volume were adjusted for intracranial volume *AD* Alzheimer’s disease, *ADAS* Alzheimer’s disease Assessment Scale, *AQT-CF* A Quick Test of cognitive speed—color and form, *CSF* cerebrospinal fluid, *MCI* mild cognitive impairment, *MMSE* Mini-Mental State Examination, *NA* not applicable, *TMT-A* Trail Making Test-A, *WML* white matter lesions

### Associations for APOE ε4 with ^18^F-AV-1451 tau PET and cortical thickness

**Fig. 1:**
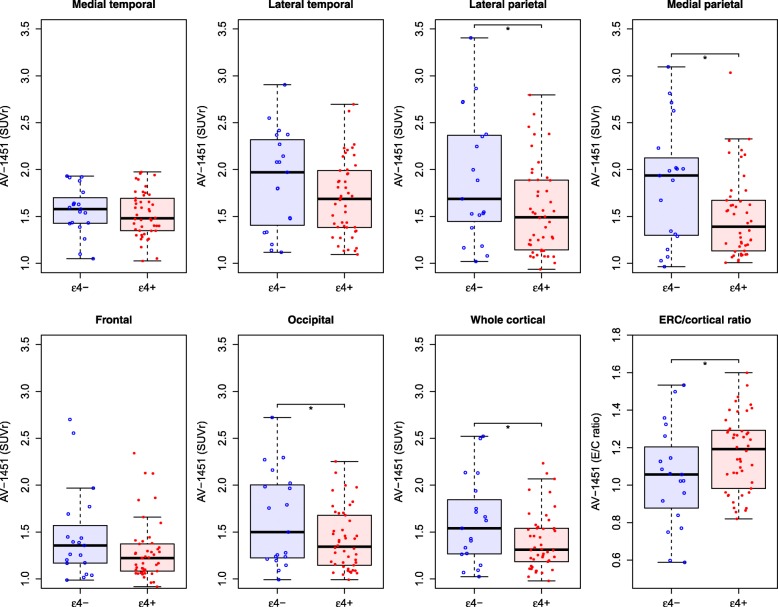
*APOE* ε4 and ^18^F-AV-1451. Regional and whole brain cortical ^18^F-AV-1451 in AD patients by *APOE* ε4 status. *P < 0.05. See Additional file 1 (Table S1) for definitions of regions, and Table 2 for *P* values in different models. SUVr, standardized uptake value ratio

**Fig. 2:**
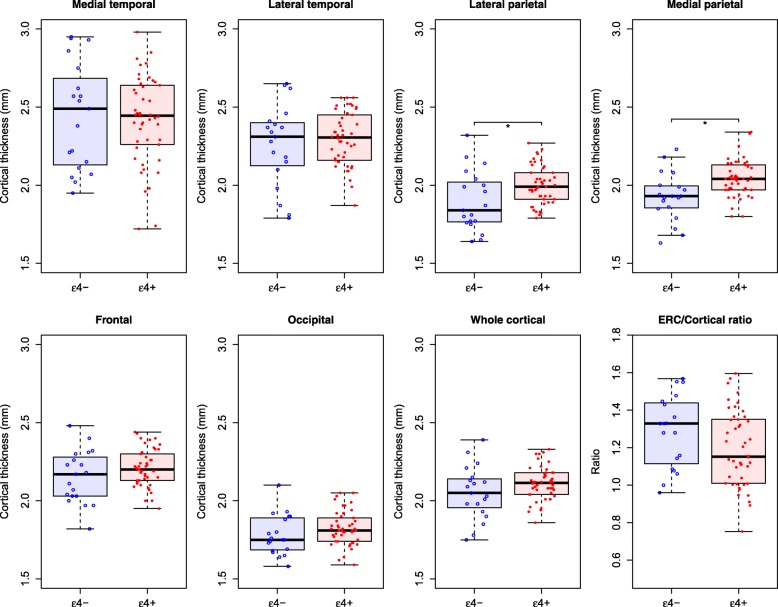
*APOE* ε4 and cortical thickness. Regional and whole brain cortical thickness in AD patients by *APOE* ε4 status. *P < 0.05. See Additional file 1 (Table S1) for definitions of regions, and Table 2 for *P* values in different models

*APOE ε4*-negative patients had greater ^18^F-AV-1451 uptake in lateral parietal, medial parietal, occipital, and whole brain cortical areas compared with *APOE ε4*-positive patients, while *APOE ε4*-positive patients had greater ^18^F-AV-1451 E/C ratio uptake (Fig. 1). All differences remained significant when adjusting for CSF Aβ42 or WML, and differences in the medial parietal cortex also remained significant when adjusting for age and sex (Table 2 and Additional file 1: Table S2). When adjusting for CSF P-tau, effects remained significant in the medial parietal cortex, with trends for the lateral parietal cortex and E/C ratio (Additional file 1: Table S2). *APOE ε4*-negative patients also had thinner cortex in lateral and medial parietal areas compared with *APOE ε4*-positive patients (Fig. 2 and Table 2).

**Table 2:** ^18^F-AV-1451 PET and cortical thickness by *APOE* status

Region	*APOE* ε4+ (unadjusted)		*APOE* ε4+ (adjusted for age and sex)		*APOE* ε4+ (adjusted for CSF Aβ42)	
	β	*P* value	β	*P* value	β	*P* value
^18^F-AV-1451						
Medial temporal	−0.039	0.561	−0.011	0.873	−0.047	0.467
Lateral temporal	−0.191	0.123	−0.128	0.269	−0.201	0.106
Lateral parietal	**−0.343**	**0.024**	−0.238	0.067	**−0.37**	**0.013**
Medial parietal	**−0.349**	**0.015**	**−0.252**	**0.044**	**−0.364**	**0.011**
Frontal	−0.175	0.092	−0.088	0.305	−0.193	0.054
Occipital	**−0.221**	**0.04**	−0.187	0.073	**−0.228**	**0.035**
Whole cortical	**−0.203**	**0.041**	−0.134	0.12	**−0.219**	**0.024**
ERC/cortex ratio	**0.123**	**0.049**	0.105	0.078	**0.128**	**0.041**
Cortical thickness						
Medial temporal	−0.022	0.787	0.01	0.899	0.015	0.875
Lateral temporal	0.037	0.496	0.05	0.38	0.046	0.475
Lateral parietal	**0.101**	**0.014**	**0.097**	**0.017**	**0.111**	**0.024**
Medial parietal	**0.116**	**0.002**	**0.113**	**0.002**	**0.125**	**0.004**
Frontal	0.056	0.137	0.057	0.144	0.065	0.137
Occipital	0.042	0.188	0.05	0.128	0.055	0.141
Whole cortical	0.057	0.104	0.063	0.083	0.07	0.093
ERC/cortex ratio	−0.090	0.102	−0.062	0.205	−0.096	0.085

Effects of *APOE* ε4+ on ^18^F-AV-1451 (top part) and cortical thickness (lower part), in different linear regression models Significant results (*P* < 0.05) are indicated in bold The unadjusted effects correspond to Figs. 1 and 2 *CSF* cerebrospinal fluid, *ERC* entorhinal cortex

In a secondary analysis, we tested effects in the AD dementia subgroup only (15 *APOE ε4*-negative and 28 *APOE ε4*-positive patients) to better account for differences in disease severity. *APOE ε4* status was not related to global cognition (MMSE (mean ± SD) 20.9 ± 5.7 vs. 20.8 ± 4.3, *P* = 0.93) or age (68.9 ± 8.1 vs. 72.1 ± 7.9, *P* = 0.18) in these patients, but *APOE ε4*-negative patients still had increased ^18^F-AV-1451 in the lateral and medial parietal and whole brain cortical areas (Additional file 1: Figure S2) and had thinner parietal cortices (Additional file 1: Figure S3).

### Interactions of APOE ε4 and ^18^F-AV-1451 tau PET to predict cortical thickness

**Fig. 3:**
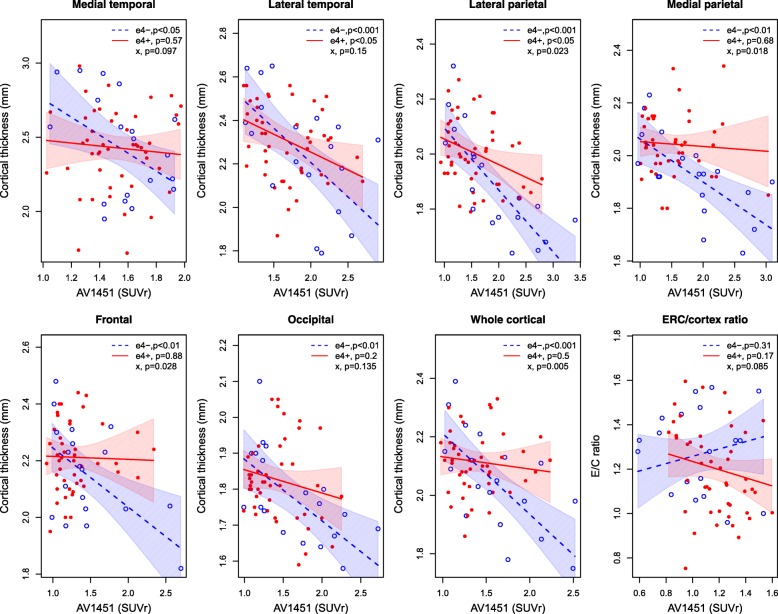
Associations between ^18^F-AV-1451 and cortical thickness in regions and in whole brain cortex. Models included the interaction between ^18^F-AV-1451 and *APOE* ε4 status. *P* values are shown for the individual *APOE* ε4 groups, and for the interaction effect. See Additional file 1 (Table S1) for definitions of regions. All models were adjusted for age and sex. SUVr, standardized uptake value ratio

Next, we tested models with interactions for *APOE* ε4 and ^18^F-AV-1451 to predict cortical thickness (Fig. 3). There were significant interactions in the lateral parietal, medial parietal, and frontal regions, and in whole brain cortex (*P* < 0.05), indicating that the effects of ^18^F-AV-1451 on cortical thickness were more pronounced in *APOE* ε4-negative than in *APOE* ε4-positive patients. The main effects of ^18^F-AV-1451 on cortical thickness were significant in *APOE* ε4-negative patients in all regions (except for the E/C ratio), but only in lateral temporal and lateral parietal regions in *APOE* ε4-positive patients.

### Effects of APOE ε4 and ^18^F-AV-1451 PET on cognition

There were no significant interactions between *APOE* ε4 and (regional or global) ^18^F-AV-1451 to predict cognitive scores. We therefore only present models without interaction terms. *APOE* ε4 was a significant predictor of worse ADAS delayed recall, and better TMT-A, while greater (whole cortical) ^18^F-AV-1451 signal was a significant predictor of worse animal fluency and (at trend level, *P* = 0.054) worse MMSE (Table 3). We next included both *APOE* ε4 and ^18^F-AV-1451 as predictors, which had minor effects on their respective separate estimates. The effect of ^18^F-AV-1451 on MMSE was β = −3.838 (*P* = 0.054) before and β = −4.024 (*P* = 0.046) after adjustment for *APOE* ε4, a difference of 4.9%. Similarly, the effect of *APOE* ε4 positivity on ADAS delayed recall was β = 2.124 (*P* = 0.01) before and β = 2.170 (*P* = 0.01) after adjustment for ^18^F-AV-1451, a difference of 2.2%. *R*^2^ for the models was only minimally affected by combining the modalities, and comparisons of AIC never favored the combination of *APOE* ε4 and ^18^F-AV-1451 over the best individual model (Table 3). Taken together, this suggests that *APOE* ε4 and ^18^F-AV-1451 had independent effects on the cognitive measures.

**Table 3:** *APOE* ε4 and ^18^F-AV-1451 to predict cognition

Cognitive test	Predictors	β-coefficients		*R* ^2^	AIC
		*APOE* ε4+	^18^F-AV-1451		
MMSE	*APOE* ε4+	−0.581 (*P* = 0.67)	NA	0.056	379.1
	^18^F-AV-1451	NA	−3.838 (*P* = 0.054)	0.112	375.2
	*APOE* ε4+ and ^18^F-AV-1451	−0.946 (*P* = 0.48)	**−4.024 (*P*** **= 0.046)**	0.12	376.6
ADAS immediate recall	*APOE* ε4+	0.352 (*P* = 0.45)	NA	0.091	211.7
	^18^F-AV-1451	NA	0.968 (*P* = 0.14)	0.118	209.9
	*APOE* ε4+ and ^18^F-AV-1451	0.426 (*P* = 0.36)	1.03 (*P* = 0.12)	0.132	211
ADAS delayed recall	*APOE* ε4+	**2.124 (*P*** **= 0.01)**	NA	0.168	260.9
	^18^F-AV-1451	NA	1.724 (*P* = 0.13)	0.086	266.3
	*APOE* ε4+ and ^18^F-AV-1451	**2.17 (*P*** **= 0.01)**	1.832 (*P* = 0.09)	0.214	259.7
TMT-A	*APOE* ε4+	**−47.961 (*P*** **= 0.03)**	NA	0.158	634.5
	^18^F-AV-1451	NA	57.62 (*P* = 0.07)	0.131	636.2
	*APOE* ε4+ and ^18^F-AV-1451	**−44.371 (*P*** **= 0.04)**	51.109 (*P* = 0.09)	0.204	633.3
Letter fluency (S)	*APOE* ε4+	2.537 (*P* = 0.11)	NA	0.167	327.1
	^18^F-AV-1451	NA	−0.659 (*P* = 0.79)	0.123	329.9
	*APOE* ε4+ and ^18^F-AV-1451	2.54 (*P* = 0.12)	0.023 (*P* = 0.99)	0.167	329.1
Animal fluency	*APOE* ε4+	1.969 (*P* = 0.27)	NA	0.108	340.1
	^18^F-AV-1451	NA	**−6.637 (*P*** **= 0.01)**	0.198	334.4
	*APOE* ε4+ and ^18^F-AV-1451	1.238 (*P* = 0.47)	**−6.304 (*P*** **= 0.02)**	0.207	335.8
AQT-CF	*APOE* ε4+	−9.364 (*P* = 0.47)	NA	0.078	518.4
	^18^F-AV-1451	NA	21.59 (*P* = 0.23)	0.097	517.3
	*APOE* ε4+ and ^18^F-AV-1451	−7.717 (*P* = 0.55)	20.403 (*P* = 0.26)	0.104	518.9

Linear regression models predicting different cognitive tests. β-coefficients are on the original scales. For each test, models were evaluated with different predictors: *APOE* ε4, ^18^F-AV-1451, or both *APOE* ε4 and ^18^F-AV-1451 (no interactions). In all cases when *APOE* ε4 or ^18^F-AV-1451were significant predictors alone, they remained significant with only minor changes in β-coefficients when adjusted for each other. All models were adjusted for age, sex and education. Comparisons of AIC never favored the combined models, since ΔAIC ranged from −1.2 to 2 for the combined model minus the best (lowest AIC) individual model (a ΔAIC ≤ 2 is generally not considered a meaningful model difference) Significant results (*P* < 0.05) are indicated in bold *ADAS* Alzheimer’s disease Assessment Scale, *AIC* Akaike information criterion, *AQT-CF* A Quick Test of cognitive speed—color and form, *MMSE* Mini-Mental State Examination, *NA* not applicable, *TMT-A* Trail Making Test-A

These models used global cortical ^18^F-AV-1451, but the results for regional ^18^F-AV-1451 were very similar (data not shown). For TMT-A, the effect of ^18^F-AV-1451 was significant in occipital, lateral parietal, and lateral temporal regions, corresponding to the trend for global ^18^F-AV-1451 (Table 3).

### Effects of APOE ε4 and ^18^F-AV-1451 PET on cognition, adjusted for cortical thickness

For cognitive tests that were associated with *APOE* ε4 (ADAS delayed recall and TMT-A) or ^18^F-AV-1451 (MMSE and animal fluency), we performed exploratory analyses with additional adjustments for brain structure.

The effects of ^18^F-AV-1451 on cognition were reduced when adjusting for cortical thickness. The association with MMSE was eliminated (*P* = 0.25) by adjusting for global cortical thickness (which had an independent significant effect; β = 12.1, *P* = 0.015). The effect on animal fluency was attenuated by adjusting for global cortical thickness (*P* = 0.055) and eliminated (*P* = 0.18) by adjusting for medial temporal lobe thickness (which had an independent significant effect; β = 6.81, *P* = 0.027).

The effects of *APOE* ε4 in this exploratory analysis were largely independent from cortical thickness. The effect on ADAS delayed recall was not affected by adjusting for global cortical thickness, and only marginally affected (β = 1.74, *P* = 0.024) by adjusting for medial temporal lobe cortical thickness (which had an independent significant effect; β = −2.97, *P* = 0.022). The effect on ADAS delayed recall also remained (β = 1.94, *P* = 0.013) when adjusting for hippocampal volume (which was not significantly associated with memory in this combined model; β = −0.0013, *P* = 0.079). For TMT-A, the effect was not affected by adjusting for global cortical thickness, and only marginally affected (β = −55.2, *P* = 0.0094) when adjusting for lateral temporal thickness (which had an independent significant effect; β = −118, *P* = 0.027).

In summary, the effects of ^18^F-AV-1451 on cognition largely depended on cortical thickness, while the effects of *APOE* ε4 on cognition were largely independent of cortical thickness and hippocampal volume.

### Associations between ^18^F-AV-1451 and demographic factors

**Fig. 4:**
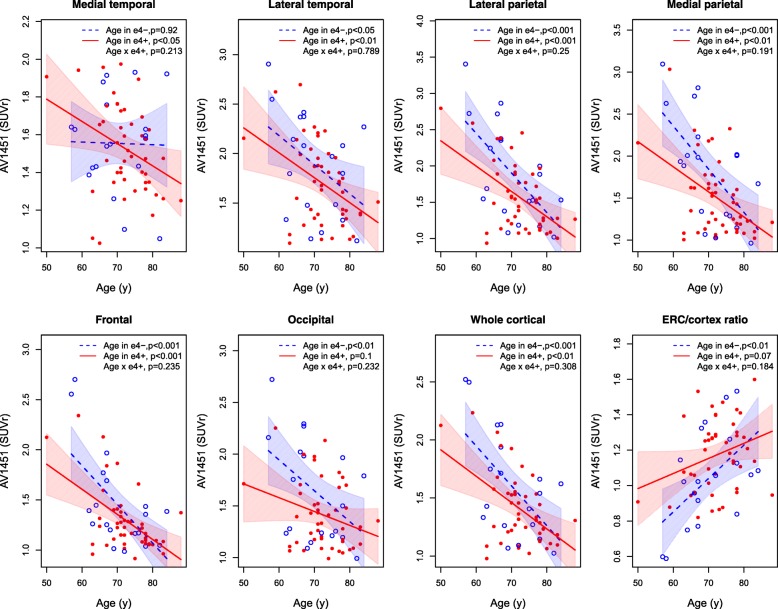
*APOE* ε4, age and ^18^F-AV-1451 PET. Regional and whole brain cortical ^18^F-AV-1451 PET in AD patients. Models included the interaction between age and *APOE* ε4 status. *P* values are shown for the individual *APOE* ε4 groups, and for the interaction effect. See Additional file 1 (Table S1) for definitions of regions. All models were adjusted for sex. SUVr, standardized uptake value ratio

Lower age was correlated with greater ^18^F-AV-1451 uptake. This was independent of *APOE ε4* status, except in the medial temporal lobe where lower age was associated with ^18^F-AV-1451 uptake in *APOE ε4*-positive but not in *APOE ε4*-negative patients (Fig. 4). There were no significant associations between sex and ^18^F-AV-1451, and no interactions between sex and *APOE ε4* status to predict ^18^F-AV-1451 (data not shown).

### Associations between ^18^F-flutemetamol and ^18^F-AV-1451

There were no significant associations between ^18^F-flutemetamol and ^18^F-AV-1451,

in either *APOE ε4*-positive or *APOE ε4*-negative patients (Additional file 1: Figure S4).

## Discussion

*APOE* ε4-negative AD patients had increased ^18^F-AV-1451 (tau) uptake and reduced cortical thickness compared with *APOE* ε4-positive patients, particularly in the parietal cortex. In contrast, *APOE* ε4-positive patients had slightly higher uptake of ^18^F-AV-1451 in the entorhinal cortex relative to the whole cortex, and also had higher cortical ^18^F-flutemetamol (Aβ) uptake. Regional associations between ^18^F-AV-1451 and cortical thickness depended on *APOE* ε4 status, as greater ^18^F-AV-1451 uptake was associated with cortical thinning mainly in *APOE* ε4-negative patients. Both ^18^F-AV-1451 and *APOE* ε4 were independently associated with cognition, but with different cognitive tests. These differences were found despite that age, sex, and global cognitive impairment not differing by *APOE* ε4 status. Taken together, these results suggest that *APOE* ε4 status influences differences in disease pathways, both through differential development and spread of tau pathology, and through effects on cognitive outcomes that may involve non-tau-related mechanisms. The proportion of *APOE* ε4-negative patients was 29%. This is in line with previous estimates of *APOE* ε4 prevalence in people with biomarker evidence of Aβ pathology, where *APOE* ε4 positivity is especially common in cohorts from northern Europe [43]. However, even at this relatively low frequency, the number of AD patients who are *APOE* ε4 negative will still be considerable in the population as a whole.

Our first main finding was that *APOE* ε4-negative patients had increased global ^18^F-AV-1451 uptake, with significant increases in the lateral parietal, medial parietal, and occipital areas. The *APOE* ε4-negative patients also had increased CSF T-tau and P-tau, which agrees with previous comparisons between ^18^F-AV-1451 and CSF tau [42, 44]. In contrast, *APOE* ε4-positive patients had higher relative ^18^F-AV-1451 uptake in the entorhinal cortex compared with the remaining cortex. Few studies have tested effects of *APOE* ε4 on tau PET imaging, but our results are consistent with a study in 62 (typical and atypical) AD patients, where the absence of *APOE* ε4 was associated with a greater ^18^F-AV-1451 signal in the neocortex and less signal in the entorhinal cortex (using a similar E/C ratio as here) [24]. A smaller study in 20 (mostly atypical) AD patients found that *APOE* ε4 was associated with greater ^18^F-AV-1451 signal in the bilateral medial temporal and right temporoparietal cortex [3]. Also, few studies have tested the effects of *APOE* ε4 on CSF tau measures when restricting the analysis to people with biomarker evidence of Aβ pathology. Altmann et al. found increased CSF tau levels in *APOE* ε4-positive controls and MCI patients [45], but since that study also included people without biomarkers supporting Aβ positivity the results are difficult to compare with our study.

Our second main finding was that *APOE* ε4-negative patients also had reduced thickness in the lateral and parietal areas (but there were no *APOE* ε4-dependent differences in hippocampal volumes). This partly agrees with results that *APOE* ε4-negative AD patients have less temporal lobe atrophy [12] and hypometabolism [46] but more frontoparietal atrophy [11, 13] compared with *APOE* ε4-positive patients. Furthermore, ^18^F-AV-1451 and *APOE* ε4 status interacted to predict cortical thickness, with stronger effects of ^18^F-AV-1451 on atrophy in *APOE* ε4-negative patients. The interaction was mainly present in the parietal and frontal lobes.

Our results support a hypothetical model in which *APOE* ε4-dependent differences in patterns of atrophy are at least partly related to differences in the spread of tau pathology. One intriguing possibility is that the cortex, with frontoparietal regions in particular, is more vulnerable to tau in patients who develop AD despite lacking *APOE* ε4. The reason for this increased vulnerability is not clear. We considered the possibility that Aβ pathology could contribute to the differences in atrophy and tau, but this is unlikely since the *APOE* ε4-positive patients had slightly greater Aβ load than the *APOE* ε4-negative patients. Previous studies have found divergent results for associations between *APOE* ε4 and Aβ in AD, with either no effect of *APOE* ε4 on Aβ pathology [47], less Aβ pathology [46, 48], or more Aβ pathology [49] in *APOE* ε4-positive AD. Potentially, patients who develop AD despite lacking *APOE* ε4 have other genetic abnormalities which put them at risk for degeneration [50]. Another possibility is that patients who develop AD and have an *APOE* ε4 allele may have partly non-tau-dependent cortical atrophy, perhaps due to impaired neuronal repair in the presence of *APOE* ε4 [51]. This may fit with the attenuated association between ^18^F-AV-1451 uptake and cortical thickness in *APOE* ε4-positive patients in this study.

The remaining part of our study was on cognition. Due to the relatively small sample size and the inherent variability of cognitive measures, these results should be interpreted with caution and be regarded as preliminary. However, we reproduced known *APOE* ε4-dependent profiles with relatively preserved memory function and more attention/processing speed impairment in *APOE* ε4-negative AD [11–14]. The fact that the associations between *APOE* ε4 status and delayed recall memory (worse in *APOE* ε4-positive) and TMT-A (worse in *APOE* ε4-negative) remained significant when adjusting for ^18^F-AV-1451 or brain atrophy suggests that the relationship between *APOE* ε4 status and cognition at least partly depends on non-tau-related mechanisms. In contrast, the associations between ^18^F-AV-1451 and cognitive tests were attenuated by adjusting for brain atrophy, which supports the hypothesis that tau accumulation leads to cognitive decline partly through atrophy [5].

Strengths of the study include the multimodal dataset, which combines brain MRI, tau and amyloid PET, CSF biomarkers, genetics, and cognitive data. Furthermore, the patients were recruited at a secondary referral clinic, which may make the sample more representative of the general AD population than samples recruited at more specialized centers. Another strength is that the *APOE ε4* groups were matched for age, sex, education, presence of dementia, and overall cognitive impairment (measured by MMSE). One limitation is the cross-sectional design. Longitudinal analyses are required to understand causal relationships between tau, atrophy, and cognition. For example, we cannot exclude the possibility of reverse causality for some of the associations, i.e., that *APOE* ε4-negative patients had premorbid thinner cortices (especially in frontoparietal regions) which predisposed them to tau accumulation. In line with this, a study on neonates found that *APOE* ε4 carriers had reductions in volumes in temporal regions, but greater volumes in the parietal, frontal, and occipital cortex compared with *APOE* ε4-negative neonates, which suggests that some *APOE* ε4-dependent differences in brain structure (and perhaps vulnerability to tau) may already be present at birth [52]. In the future, it will be informative to extend the current study with longitudinal data.

## Conclusions

*APOE ε4* status is associated with heterogeneity not only in clinical presentation and atrophy patterns in AD, but also in the accumulation of tau pathology. *APOE ε4*-negative patients may be more likely to deposit tau and have more atrophy in the parietal lobes compared with *APOE ε4*-positive AD, which predispose them to a nonamnestic clinical phenotype.

## Additional file

Additional file 1: Supplementary material. (DOCX 286 kb)
