# Supplementary material for: Greater tau load and reduced cortical thickness in APOE ε4-negative Alzheimer’s disease: a cohort study
Source: Alzheimers Res Ther. 2018 Aug 7;10:77. doi: 10.1186/s13195-018-0403-x (PMC6081879; doi:10.1186/s13195-018-0403-x)
Supplement: Supplementary file 1 — Supplementary material. (DOCX 286 kb) [file 13195_2018_403_MOESM1_ESM.docx]

***Additional file1***

**Greater tau load and reduced cortical thickness in *APOE* ε4-negative Alzheimer’s disease: a cohort study**

Niklas Mattsson§^1,2^, Rik Ossenkoppele§^1,3^, Ruben Smith^1,2^, Olof Strandberg^1^, Tomas Ohlsson^4^, Jonas Jögi^5^, Sebastian Palmqvist^1,2^, Erik Stomrud^1^, Oskar Hansson^1,6^

§These authors contributed equally

^1^Clinical Memory Research Unit, Department of Clinical Sciences, Faculty of Medicine, Lund University, Lund/Malmö, Sweden

^2^Department of Neurology, Skåne University Hospital, Lund, Sweden

^3^VU University Medical Center, Neuroscience Campus Amsterdam, Amsterdam, the Netherlands

^4^Department of Radiation physics, Skåne University Hospital, Lund, Sweden

^5^Department of Clinical Physiology and Nuclear Medicine, Skåne University Hospital, Lund, Sweden

^6^Memory Clinic, Skåne University Hospital, Malmö, Sweden

**Table S1. Definitions of meta-regions for MRI and 18F-AV-1451**

| **Combined Region** | **FreeSurfer Labels** | **FreeSurfer Label Names** |
| --- | --- | --- |
| Frontal | 1003, 1012, 1014, 1018, 1019, 1020, 1027, 1028, 1032, 2003, 2012, 2014, 2018, 2019, 2020, 2027, 2028, 2032 | ctxcaudalmiddlefrontal, ctx-lh-lateralorbitofrontal, ctx-lh-medialorbitofrontal, ctx-lh-parsopercularis, ctx-lh-parsorbitalis, ctx-lh-parstriangularis, ctx-lh-rostralmiddlefrontal, ctx-lh-superiorfrontal, ctx-lh-frontalpole, ctx-rh-caudalmiddlefrontal, ctx-rh-lateralorbitofrontal, ctx-rh-medialorbitofrontal, ctx-rh-parsopercularis, ctx-rh-parsorbitalis, ctx-rh-parstriangularis, ctx-rh-rostralmiddlefrontal, ctx-rh-superiorfrontal, ctx-rh-frontalpole |
| Lateral Temporal | 1001, 1009, 1015, 1030, 1034, 2001, 2009, 2015, 2030, 2034 | ctx-lh-bankssts, ctx-lh-inferiortemporal, ctx-lh-middletemporal, ctx-lh-superiortemporal, ctx-lh-transversetemporal, ctx-rh-bankssts, ctx-rh-inferiortemporal, ctx-rh-middletemporal, ctx-rh-superiortemporal, ctx-rh-transversetemporal |
| Lateral Parietal | 1008, 1029, 1031, 2008, 2029, 2031 | ctx-lh-inferiorparietal, ctx-lh-superiorparietal, ctx-lh-supramarginal, ctx-rh-inferiorparietal, ctx-rh-superiorparietal, ctx-rh-supramarginal |
| Medial Parietal | 1010, 1025, 2010, 2025 | ctx-lh-isthmuscingulate, ctx-lh-precuneus, ctx-rh-isthmuscingulate, ctx-rh-precuneus |
| Occipital | 1005, 1011, 1013, 1021, 2005, 2011, 2013, 2021 | ctx-lh-cuneus, ctx-lh-lateraloccipital, ctx-lh-lingual, ctx-lh-pericalcarine, ctx-rh-cuneus, ctx-rh-lateraloccipital, ctx-rh-lingual, ctx-rh-pericalcarine |
| Medial Temporal | 1006, 1016, 2006, 2016 | ctx-lh-entorhinal, ctx-lh-parahippocampal, ctx-rh-entorhinal, ctx-rh-parahippocampal |
| Cortical | 1001, 1002, 1003, 1005, 1006, 1007, 1008, 1009, 1010, 1011, 1012, 1013, 1014, 1015, 1016, 1017, 1018, 1019, 1020, 1021, 1022, 1023, 1024, 1025, 1026, 1027, 1028, 1029, 1030, 1031, 1032, 1033, 1034, 1035, 2001, 2002, 2003, 2005, 2006, 2007, 2008, 2009, 2010, 2011, 2012, 2013, 2014, 2015, 2016, 2017, 2018, 2019, 2020, 2021, 2022, 2023, 2024, 2025, 2026, 2027, 2028, 2029, 2030, 2031, 2032, 2033, 2034, 2035 | ctx-lh-bankssts, ctx-lh-caudalanteriorcingulate, ctx-lh-caudalmiddlefrontal, ctx-lh-cuneus, ctx-lh-entorhinal, ctx-lh-fusiform, ctx-lh-inferiorparietal, ctx-lh-inferiortemporal, ctx-lh-isthmuscingulate, ctx-lh-lateraloccipital, ctx-lh-lateralorbitofrontal, ctx-lh-lingual, ctx-lh-medialorbitofrontal, ctx-lh-middletemporal, ctx-lh-parahippocampal, ctx-lh-paracentral, ctx-lh-parsopercularis, ctx-lh-parsorbitalis, ctx-lh-parstriangularis, ctx-lh-pericalcarine, ctx-lh-postcentral, ctx-lh-posteriorcingulate, ctx-lh-precentral, ctx-lh-precuneus, ctx-lh-rostralanteriorcingulate, ctx-lh-rostralmiddlefrontal, ctx-lh-superiorfrontal, ctx-lh-superiorparietal, ctx-lh-superiortemporal, ctx-lh-supramarginal, ctx-lh-frontalpole, ctx-lh-temporalpole, ctx-lh-transversetemporal, ctx-lh-insula, ctx-rh-bankssts, ctx-rh-caudalanteriorcingulate, ctx-rh-caudalmiddlefrontal, ctx-rh-cuneus, ctx-rh-entorhinal, ctx-rh-fusiform, ctx-rh-inferiorparietal, ctx-rh-inferiortemporal, ctx-rh-isthmuscingulate, ctx-rh-lateraloccipital, ctx-rh-lateralorbitofrontal, ctx-rh-lingual, ctx-rh-medialorbitofrontal, ctx-rh-middletemporal, ctx-rh-parahippocampal, ctx-rh-paracentral, ctx-rh-parsopercularis, ctx-rh-parsorbitalis, ctx-rh-parstriangularis, ctx-rh-pericalcarine, ctx-rh-postcentral, ctx-rh-posteriorcingulate, ctx-rh-precentral, ctx-rh-precuneus, ctx-rh-rostralanteriorcingulate, ctx-rh-rostralmiddlefrontal, ctx-rh-superiorfrontal, ctx-rh-superiorparietal, ctx-rh-superiortemporal, ctx-rh-supramarginal, ctx-rh-frontalpole, ctx-rh-temporalpole, ctx-rh-transversetemporal, ctx-rh-insula |

**Table S2. Effects of *APOE* ε4 when adjusting for WML or CSF P-tau**

| **Region** | ***APOE* ε4+**  **(adj. for WML)** | | ***APOE* ε4+**  **(adj. for CSF P-tau)** | |
| --- | --- | --- | --- | --- |
|  | **β** | **P-value** | **β** | **P-value** |
| *^18^F-AV-1451* | | | | |
| Medial temporal | -0.050 | 0.455 | 0.012 | 0.850 |
| Lateral temporal | -0.193 | 0.124 | -0.110 | 0.380 |
| Lateral parietal | **-0.351** | **0.023** | -0.281 | 0.061 |
| Medial parietal | **-0.360** | **0.013** | **-0.295** | **0.046** |
| Frontal | -0.171 | 0.104 | -0.109 | 0.275 |
| Occipital | **-0.214** | **0.049** | -0.116 | 0.261 |
| Whole cortical | **-0.204** | **0.044** | -0.140 | 0.148 |
| ERC/cortex ratio | 0.117 | 0.062 | 0.121 | 0.067 |
|  |  |  |  |  |
| *Cortical thickness* | | | | |
| Medial temporal | -0.043 | 0.596 | -0.053 | 0.539 |
| Lateral temporal | 0.016 | 0.757 | 0.042 | 0.472 |
| Lateral parietal | **0.097** | **0.019** | **0.098** | **0.025** |
| Medial parietal | **0.111** | **0.003** | **0.12** | **0.002** |
| Frontal | 0.045 | 0.211 | 0.055 | 0.166 |
| Occipital | 0.037 | 0.244 | 0.042 | 0.209 |
| Whole cortical | 0.047 | 0.168 | 0.055 | 0.146 |
| ERC/cortex ratio | -0.096 | 0.084 | -0.104 | 0.075 |

Effects of *APOE* ε4+ on ^18^F-AV-1451 (top part) and cortical thickness (lower part), in different linear regression models, adjusting for WML or CSF P-tau. P<0.05 indicated in bold. ERC, entorhinal cortex.

**Figure S1. *APOE* ε4 and ^18^F-flutemetamol**

Regional and whole brain cortical ^18^F-flutemetmol in AD patients by *APOE* ε4 status. * indicates P<0.05. See Table S1 for definitions of regions. The differences remained significant when adjusting for age and sex.

**Figure S2. *APOE* ε4 and ^18^F-AV-1451 in AD dementia patients**

Regional and whole brain cortical ^18^F-AV-1451 in AD dementia patients by *APOE* ε4 status. *P<0.05. See Table S1 for definitions of regions.

**Figure S3. *APOE* ε4 and cortical thickness in AD dementia patients**

Regional and whole brain cortical thickness in AD dementia patients by *APOE* ε4 status. *P<0.05. See Table S1 for definitions of regions.

**Figure S4. ^18^F-flutemetamol and ^18^F-AV-1451**

Regional and whole brain cortical ^18^F-flutemetmol and ^18^F-AV-1451 in AD patients by *APOE* ε4 status. See Table S1 for definitions of regions. The models were adjusted for age and sex. There were no significant associations between ^18^F-flutemetmol and ^18^F-AV-1451.
